# New roles for autophagy and spermidine in T cells

**DOI:** 10.15698/mic2015.03.195

**Published:** 2015-03-02

**Authors:** D. J. Puleston, A. K. Simon

**Affiliations:** 1 MRC Human Immunology Unit, Weatherall Institute of Molecular Medicine, University of Oxford, OX3 9DS.

**Keywords:** autophagy, T cell memory, spermidine, T cells, ageing, immunosenescence, immunity

## Abstract

The conserved lysosomal degradation pathway autophagy is now recognised as an essential cog in immune function. While functionally widespread in the innate immune system, knowledge of its roles in adaptive immunity is more limited. Although autophagy has been implicated in naïve T cell homeostasis, its requirement in antigen-specific T cells during infection was unknown. Using a murine model where the essential autophagy gene *Atg7* is deleted in the T cell lineage, we have shown that autophagy is dispensable for effector CD8^+^ T cell responses, but crucial for the formation of memory CD8^+^ T cells. Here, we suggest reasons why autophagy might be important for the formation of long-lasting immunity. Like in the absence of autophagy, T cell memory formation during ageing is also defective. We observed diminished autophagy levels in T cells from aged mice, linking autophagy to immunosenescence. Importantly, T cell responses to influenza vaccination could be significantly improved using the autophagy-inducing compound spermidine. These results suggest the autophagy pathway as a desirable target to improve aged immunity and modulate T cell function.

In recent times, the number of studies on macroautophagy have substantially increased. Macroautophagy, herein referred to as autophagy, is a major lysosomal degradation pathway. Its importance for eukaryotic life is not only highlighted by its conserved nature, preserved from yeast to mammals, but also in the increasing array of cellular functions autophagy can perform. This is especially true in the immune system, where autophagy has been implicated in bacterial handling, phagocytosis, cytokine production, and the downstream functions of pattern recognition receptors, to name but a few. But while autophagy is relatively well described in the innate immune system, its role in adaptive immunity is less clear. However, recent articles have detailed a role for autophagy in B cell memory formation, the regulation of antibody production and plasma cell survival. While it was known that autophagy was important for naïve T cell homeostasis, its role during the T cell response to infection was unknown. Following infection, antigen specific T cells undergo rapid proliferation, giving rise to a population of effector cells that eventually contract, leaving behind a small, but long-lived population of memory T cells that can respond rapidly to secondary infection. We showed in a recent publication that autophagy was in fact crucial for the formation of memory CD8^+^ T cells. Using a mouse model where the essential autophagy gene *Atg7* is deleted in T cells under the *CD4*-promoter (T-*Atg7^-/-^* mice), we showed that the CD8^+^ T cell effector phase proceeds in a normal fashion in the absence of autophagy to influenza and murine cytomegalovirus (MCMV) infection. However, during contraction, the *Atg7^-/-^* effector CD8^+^ T cell (CD8^+^ T_eff_) pool undergoes a catastrophic collapse, resulting in the failure to form the CD8^+^ T cell memory compartment. *Atg7^-/-^* antigen-specific CD8^+^ T_eff_ cells appear to be phenotypically normal, both transcriptionally and with markers at the cell surface. However, such cells boast a high mitochondrial burden compared to wild type antigen-specific CD8^+^ T cells alongside an increase in mitochondrial reactive oxygen species (ROS). Work from Erika Pearce’s group has demonstrated how mitochondrial biogenesis takes place during the CD8^+^ T cell effector phase in response to IL-15 as cells begin to switch to mitochondrial respiration, an important event for memory CD8^+^ T cell formation. Thus, prior to memory formation this high mitochondrial load, coupled to the kick-start of oxidative phosphorylation (OXPHOS), would lead to a flood of electrons being shed from the electron transport chain that can interact with molecular oxygen resulting in superoxide production. Therefore, mitochondria are likely to require strict regulation during the late effector stages of the CD8^+^ T cell response to which autophagy is known to contribute substantially. As a result, we hypothesised that increased ROS production due to dysregulated mitochondrial homeostasis in the absence of autophagy might be driving the collapse of the antigen-specific effector pool in CD8^+^ T cells responding to infection. Indeed, in more recent unpublished observations, we found the CD8^+^ T cell memory compartment could be rescued in T-*Atg7^-/-^* mice following *in vivo* administration of the antioxidant compound N-acetyl cysteine. We therefore put forward a model where autophagy is an essential antioxidant pathway in antigen-specific CD8^+^ T cells, preventing excess ROS production and apoptosis through the regulation of mitochondrial load and quality (Figure 1).

**Figure 1:**
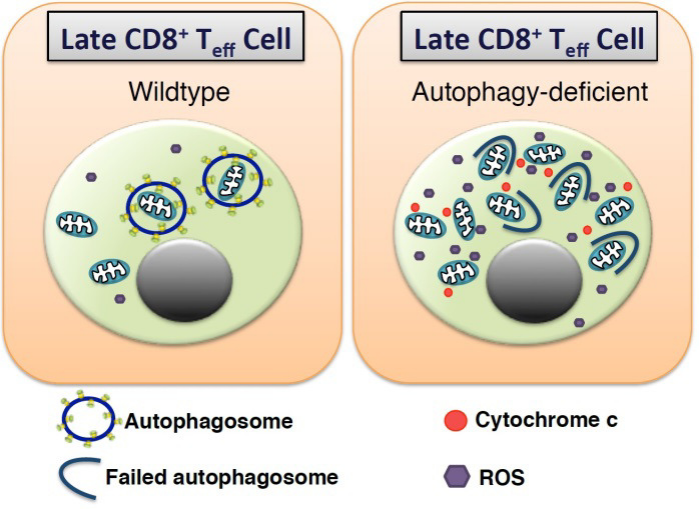
FIGURE 1: Autophagy acts as an antioxidant pathway in antigen-specific CD8^+^ T cells. During the effector phase in wild type mice, autophagy regulates mitochondrial load, acting to maintain organelle quality and limiting ROS production. In the absence of autophagy, the mitochondrial burden of late effector CD8^+^ T cells (CD8^+^ T_eff_) is increased, quality control is diminished, both of which contribute to excess ROS formation resulting in cell death.

As previously mentioned, metabolism plays a key role in regulating CD8^+^ T cell differentiation. Following activation, cells switch to glycolysis in order to support cell proliferation. Subsequently during contraction, memory precursor cells turn to OXPHOS, particularly fatty acid oxidation (FAO), which facilitates memory CD8^+^ T cell differentiation. We attempted to investigate the metabolic profile of autophagy-deficient CD8^+^ T_eff_ cells by measuring glucose transporter (GLUT)-1 expression at various stages of CD8^+^ T cell differentiation. In wild type CD8^+^ T cells, GLUT-1 is upregulated following activation to support glycolysis and is then downregulated in late-stage CD8^+^ T_eff_ cells as they begin to use both glycolysis and OXPHOS. Autophagy-deficient CD8^+^ T_eff_ displayed significantly greater GLUT-1 expression at the peak of the effector phase and GLUT-1 downregulation did not occur in late-effector phase like it did in wild type mice. Similar to this, we have found in unpublished experiments that glucose uptake is also significantly increased in *Atg7^-/-^* early and late CD8^+^ T_eff_ cells relative to wild type controls. These results imply a situation of sustained glycolysis in *Atg7^-/-^* CD8^+^ T_eff_ cells that might reflect a failure to switch to mitochondrial respiration. Recent work, again from Erika Pearce’s group, has detailed how CD8^+^ T_eff_ cells undergo cell-intrinsic lipolysis to provide fatty acid intermediates for fatty acid oxidation (FAO), an essential step for memory formation. In new unpublished work, we have observed significantly increased lipid content in autophagy-deficient CD8^+^ T_eff_ cells with altered fatty acid uptake compared to wild type cells. These data have led us to posit an additional model, where autophagy is required for the switch to FAO during memory formation through the delivery of lipid stores to the lysosome where they are broken down by lysosomal acid lipase (Figure 2). In the absence of autophagy, CD8^+^ T_eff_ cells are unable to switch efficiently to mitochondrial respiration due to a failure to provide fatty acid metabolites for FAO, thus cells remain in a prolonged state of glycolysis. That leads to metabolic instability, energy crisis, and possibly apoptosis. What induces autophagy during the effector phase to mediate these important functions is unclear. Perhaps factors required for memory formation such as IL-15 or the withdrawal of other growth factors, such as IL-2, regulate autophagy induction. Alternatively, changes in metabolite profile during the CD8^+^ T_eff_ phase might signal to the nutrient sensors AMPK and mTOR, which also act as major regulators of autophagy.

**Figure 2:**
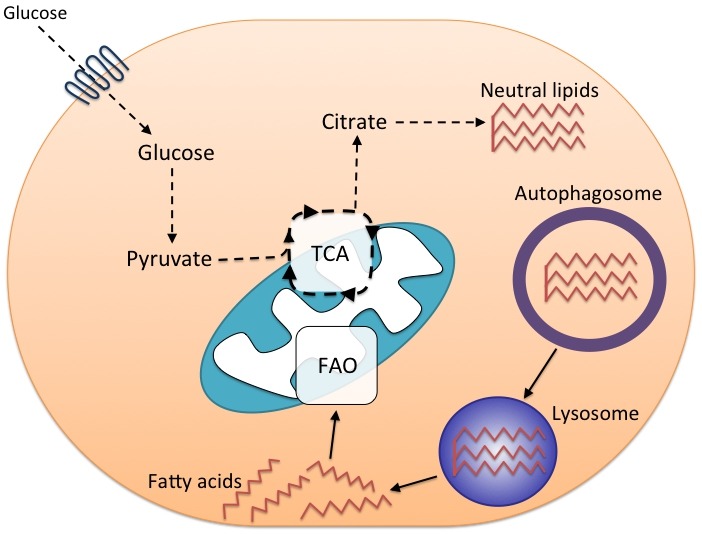
FIGURE 2: Autophagy and fatty acid metabolism are important for memory CD8^+^ T cell formation. During contraction, memory precursor cells must switch to the oxidation of fats to maintain viability. Extracellular glucose is used to fuel lipid synthesis from tricarboxylic acid cycle (TCA) precursors. In this model, autophagy is then required to sequester neutral lipid stores in autophagosomes in order to deliver them to the lysosome for degradation. The resulting free fatty acids can then enter the mitochondria for fatty acid oxidation (FAO) and energy production.

A large body of evidence has come to the fore since the turn of the century linking autophagy to the ageing process. In mice and other lower organisms, induction of autophagy or removal of autophagy genes either extends or diminishes life-span, respectively. Tellingly, the pro-longevity effects of calorie restriction are lost when autophagy genes are knocked out or knocked down in *C. elegans*. While low autophagy levels have been linked to the appearance of age-related phenomena in a number of tissues, the role of autophagy in immune ageing (termed immunosenescence) has been little studied. During ageing, defects in the T cell response become apparent. Elderly mice and humans exhibit altered T cell expansion and memory formation to both infection and vaccination, likely increasing disease susceptibility and reducing vaccine efficacy. In our recent publication, we showed that autophagy levels are diminished in both polyclonal and influenza-specific CD8^+ ^T cells from aged mice. Interestingly, the CD8^+^ T cell response of aged mice to influenza vaccination and infection could be dramatically improved when mice were treated with the autophagy-inducing compound spermidine. This effect was lost in aged T-*Atg7^-/-^* mice, which are autophagy-deficient in the T cell lineage. This suggests falling autophagy levels are a cell-intrinsic driving force behind T cell immunosenescence and might contribute to the observed defects in T cell memory formation commonly observed in elderly humans. Falling T cell autophagy levels in the aged might possibly have a similar effect to what is observed during autophagy-deletion in T-*Atg7^-/-^* mice. In line with the models proposed here, low autophagy levels would increase ROS production as a result of poor mitochondrial homeostasis and reduce lipolysis and fatty acid formation due to decreased delivery of lipid stores to lysosomes. This would have the effect of increased energetic instability and susceptibility to apoptosis resulting in fewer cells entering the memory pool in the elderly. Crucially, our results open the door to the prospect of T cell modulation through autophagy. Immunomodulators with the ability to improve T cell responses are limited, but their need are becoming ever more important as we realise the power of T cells in fighting cancer and prominent infections such as malaria, HIV and pandemic influenza. Furthermore, with the ageing population scheduled to grow significantly in the coming decades, ways to improve aged immunity and vaccine efficacy in the elderly are of great importance, targeting the autophagy pathway with compounds like spermidine might provide a great opportunity to achieve this.

